# Anisotropic Thermal Conductivity in Pellet-Based 3D-Printed Polymer Structures for Advanced Heat Management in Electrical Devices

**DOI:** 10.3390/polym18010093

**Published:** 2025-12-29

**Authors:** Michal Rzepecki, Andrzej Rybak

**Affiliations:** ABB Corporate Technology Center, Starowislna 13A, 31-038 Krakow, Poland; michal.rzepecki@pl.abb.com

**Keywords:** thermal conductivity, additive manufacturing, polymer composites, anisotropic properties, pellet printing, filler orientation, thermal management

## Abstract

Efficient thermal management is critical for modern electrical and electronic devices, where increasing power densities and miniaturization demand advanced heat dissipation solutions. This study investigates anisotropic thermal conductivity in polymer structures fabricated via pellet-based fused granulate fabrication using polyamide 6 composite filled with thermally conductive, electrically insulative mineral fillers. Three sample orientations were manufactured by controlling the printing path direction to manipulate filler alignment relative to heat flow. The microscopic analysis confirmed a flake-shaped filler orientation dependent on extrusion direction. Thermal conductivity measurements using a guarded heat flow meter revealed significant anisotropy: samples with fillers aligned parallel to heat flow exhibited thermal conductivity of 4.09 W/m·K, while perpendicular alignment yielded 1.21 W/m·K, representing a 238% enhancement and an anisotropy ratio of 3.4. The dielectric measurements showed modest electrical anisotropy with maintained low dielectric loss below 0.05 at 1 kHz, confirming the suitability of the investigated materials for electrical insulation applications. The presented results demonstrate that pellet-based fused granular fabrication uniquely enables in situ control of platelet filler orientation during printing, achieving unprecedented thermal anisotropy, high through-plane thermal conductivity, and excellent electrical insulation in directly 3D-printed polymer structures, offering a breakthrough approach for advanced thermal management in electrical devices.

## 1. Introduction

The miniaturization and integration of electronic components have significantly increased power densities, making thermal management a critical design consideration for modern electrical and electronic devices. Inefficient heat dissipation leads to localized hotspots, accelerated aging, and ultimately device malfunction or catastrophic failure. Studies indicate that thermal failure accounts for the majority of electronic device breakdowns, underscoring the importance of effective heat management strategies to ensure reliability and longevity [1,2,3,4]. As devices become smaller and operate at higher frequencies, conventional cooling solutions, such as heat sinks and fans, often fall short, necessitating advanced materials and architectures capable of directing heat away from sensitive components [5,6,7].

Polymers are widely used in electrical insulation due to their excellent dielectric properties; however, their inherently low thermal conductivity (typically ca. 0.2 W·m^−1^·K^−1^) poses a challenge for heat management. To overcome this limitation, extensive research has focused on incorporating thermally conductive fillers into polymer matrices. Carbon-based additives, such as graphite, graphene nanoplatelets, and carbon nanotubes, dominate the literature due to their exceptional intrinsic thermal conductivity [8,9,10]. However, these fillers are electrically conductive, which restricts their use in applications requiring electrical insulation. Recent studies have addressed this issue by developing polymer composites filled with ceramic particles, such as hexagonal boron nitride (h-BN), which provide high thermal conductivity while maintaining electrical insulation [11,12,13]. For example, boron nitride-filled polyethylene composites demonstrated significant improvements in thermal conductivity without compromising dielectric strength, making them promising candidates for electrical insulation components in power devices [11]. Core-shell filler strategies and alignment techniques have also been investigated to further enhance thermal performance [14,15,16].

Additive manufacturing (AM), commonly known as 3D printing, has emerged as a transformative technology for producing complex geometries and multifunctional components without the constraints of traditional subtractive methods. AM enables the fabrication of lightweight structures with tailored thermal properties, offering unprecedented design freedom for thermal management applications [17,18,19]. Among AM techniques, pellet extrusion-based 3D printing, also referred to as fused granulate fabrication (FGF), is gaining attraction due to its ability to process industrial-grade polymers and composites at reduced cost and higher throughput compared to filament-based methods. Pellet extrusion offers several advantages, including material versatility, cost efficiency, scalability, and sustainability [20,21,22,23]. These benefits make pellet extrusion particularly attractive for manufacturing functional structures aimed at thermal management in electrical devices.

Recent advances in the AM of thermally conductive polymer composites have demonstrated significant progress in achieving enhanced heat dissipation for electronics and thermal management applications. Bagatella et al. developed 3D-printable flexible resin composites with unmodified boron nitride platelets using vat photopolymerization (VPP), achieving thermal conductivity up to 0.73 W/m·K with modest anisotropy ratio of 1.4 at 20 wt.% BN loading [24]. Their work demonstrated the feasibility of maintaining electrical insulation while enhancing thermal transport in flexible electronics. Blanco et al. investigated PLA-based composites with milled carbon fibers fabricated via fused filament fabrication (FFF), reporting thermal conductivity in the range of 0.16 ÷ 0.20 W/m·K [25]. However, carbon-based fillers introduce electrical conductivity, limiting their applicability in electrical insulation. Ji et al. provided a comprehensive review of thermal management via additive manufacturing, highlighting the potential of AM to create complex geometries and functionally graded structures for heat dissipation [9].

Despite these advances, the majority of research has concentrated on isotropic thermal conductivity improvements using carbon-based fillers. There is a notable lack of studies on 3D-printed polymer structures that combine high thermal conductivity, electrical insulation, and directional (anisotropic) heat transfer. For certain applications, such as localized cooling of high-power components or thermal routing in confined spaces, the anisotropic heat conduction is essential to guide heat flow along preferred directions while minimizing undesired thermal spreading [26]. Recent work on anisotropic thermal conductivity in 3D-printed composites has primarily focused on carbon-filled systems, which are unsuitable for electrically insulating applications [25,27,28]. These studies demonstrate the feasibility of tuning anisotropy through extrusion-based printing; however, these approaches have yet to be extended to electrically insulating systems.

Therefore, the present study aims to address this gap. The novelty of this work lies in three key aspects: (i) achieving a high thermal anisotropy ratio for electrically insulating 3D-printed thermoplastic composites; (ii) demonstrating that pellet-based fused granulate fabrication can control filler orientation to engineer directional heat transfer while maintaining electrical insulation, a combination rarely achieved in additive manufacturing; and (iii) providing the systematic investigation of orientation-dependent thermal and dielectric properties in pellet-extruded PA6 composites with mineral filler, which is directly relevant for thermal management components in electrical devices.

## 2. Materials and Methods

### 2.1. Polymer Pellets

The pellets of LNP KONDUIT Compound PX13012 (SABIC, Riyadh, Saudi Arabia) was used in the 3D-printing process. This polymer composite is based on polyamide 6 (PA 6) resin containing a proprietary thermally conductive and electrically insulative mineral filler. It also contains a non-brominated, non-chlorinated flame retardant. This system is mainly processed by injection molding and is used in electrical and electronics applications for thermal management and heat dissipation, e.g., LED lighting housing, and heat sinks. The selected properties of the raw material are presented in Table 1.

### 2.2. Manufacturing of Printed Structures

The selected manufacturing method—Fused Granulate Fabrication (FGF) is characterized by freedom in relation to thermoplastic materials selection. What distinguishes it from fused filament fabrication (FFF) is the shape of the used raw materials. For FFF, it is necessary to extrude filaments with high diameter accuracy, which is challenging, especially for brittle materials. Considering this, the greatest advantage of FGF is the possibility of using raw materials in the form of granulates, which is a base form of thermoplastic materials offered by most suppliers. This feature makes it possible to use LNP KONDUIT Compound PX13012 material (SABIC, Riyadh, Saudi Arabia), which has a tensile strain-at-break level of 1.1% in extrusion-based 3D printing.

**Figure 1:**
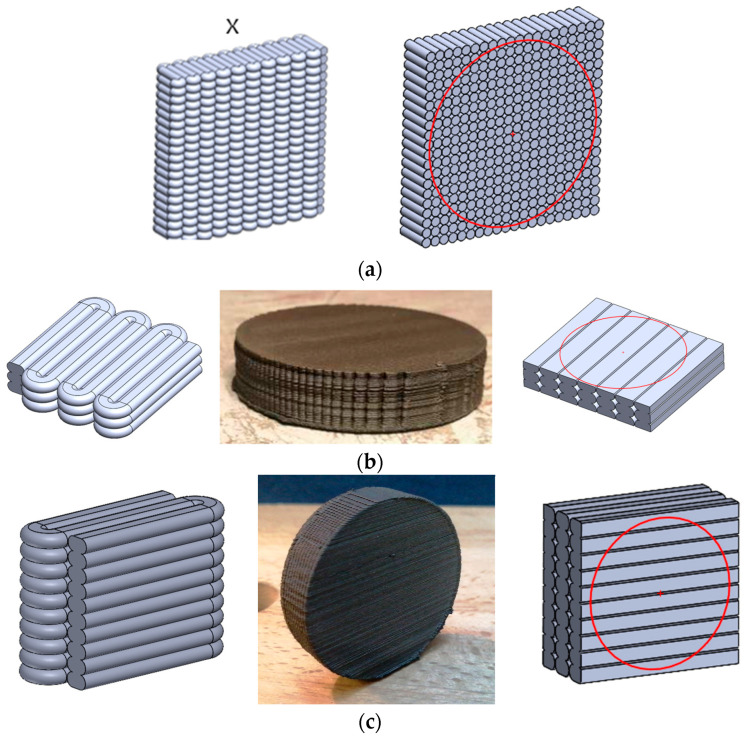
Schemes showing the course of the extruded line during printing, photos of the printed samples, and schemes of the polished samples with the microscopic observation area marked by red circle and the direction of thermal and dielectric conductivity measurements: (**a**) sample X; (**b**) sample Y; and (**c**) sample Z.

Three types of samples were manufactured by means of FGF technology (Pollen AM, Ivry-sur-Seine, France) with the geometry of the disc having a diameter of 55 mm and a thickness of 12 mm. The main difference between the samples was the direction of the majority length of the extruder path, as shown in Figure 1. Sample “X” was printed in such a direction that the majority of the path length was perpendicular to the flat surface of the disc. Sample “Y” was printed in such a direction that the direction of the layer growth was perpendicular to the flat surface of disc. Sample “Z” was printed in such a direction that both the direction of the majority length of the extruder path and the direction of layer growth were parallel to the flat surface of disc. To reduce the influence of areas where the extruder path was different than desired, samples were machined to disc with a 50 mm diameter and a 10 mm thickness. The measured samples thus had uniform direction of material flow for the whole volume of the sample.

### 2.3. Observation of Printed Structures

Observations of the printed structures were carried out using a digital optical microscope, Keyence VHX-6000 (Keyence, Osaka, Japan). Prior to imaging, all samples were carefully polished to remove surface irregularities and subsequently cleaned to eliminate residual contaminants. This preparation ensured high-quality visualization of the microstructural features and accurate assessment of the surface morphology. The observed plane for each sample was perpendicular to the direction of heat flow during the thermal conductivity measurement. Thanks to the known and predictable production process, it was possible to make the assumption that images of the surfaces of sample Y and Z represented two cross-sections of sample X, that images of the surface of sample X and Z represented two cross-sections of sample Y, and, respectively, images of the surfaces of sample X and Y represented two cross-sections of sample Z.

### 2.4. Thermal Conductivity Measurement

Thermal conductivity measurements were carried out using a Guarded Heat Flow Meter GHFM-02 (Thermtest, Veddige, Sweden) in compliance with ASTM E1530-19 [29]. This method is designed for solids such as metals, polymers, and composites with low to medium thermal conductivity. The technique is based on a steady-state heat flow approach, where the sample is subjected to a controlled through-thickness temperature gradient. Heat is applied via an upper heater plate and removed through a lower heat sink plate. A guard heater surrounds the main heater to minimize lateral heat losses, ensuring predominantly one-dimensional heat transfer. Samples were machined to the required dimensions of 50 mm diameter and 10 mm thickness and cleaned to remove surface contaminants. A thin layer of thermal interface paste was applied to both faces to improve thermal contact and reduce interfacial resistance. The sample was placed between the heater and heat sink plates within the guarded zone. The guard heater was actively controlled to match the temperature of the main heater, reducing radial heat flow. The assembly was enclosed in an insulated chamber to minimize environmental losses. The system was allowed to reach thermal equilibrium, indicated by stable temperature readings at all sensors. Heat flux through the sample was measured using a calibrated heat flow transducer. Temperatures were recorded at the upper and lower surfaces of the sample, along with an additional reference temperature. Thermal conductivity (*λ*) was calculated using Fourier’s law:

(1) $$\lambda =\frac{q\cdot d}{\Delta T}\left(\frac{W}{m\cdot K}\right),$$

where q is the heat flux (W·m^−2^), d is the sample thickness (*m*), and ΔT is the temperature difference across the sample (*K*).

Thermal resistance was calculated as:

(2) $$R=\frac{d}{\lambda }\left(\frac{m^{2}\cdot K}{W}\right).$$

Thermal conductivity measurement settings and conditions are listed in Table 2.

### 2.5. Dielectric Measurement

Broadband dielectric measurements were performed using a Novocontrol Turnkey Broadband Dielectric Spectrometer (Novocontrol Technologies GmbH & Co. KG, Montabaur, Germany). The system allows for the frequency-dependent characterization of permittivity over a wide range of frequencies by employing a high-precision impedance analyzer integrated with a temperature-controlled sample chamber. The spectrometer operates under a fully automated control environment, ensuring accurate measurement of dielectric properties, such as permittivity (ε′), dielectric loss (ε″), and conductivity, as functions of frequency and temperature. Samples were cut off from 3D-printed discs with high precision and received samples for broadband dielectric measurements that had the form of discs with a 21 mm diameter and a 2 mm thickness. Samples with the Z printing orientation broke during samples preparation, which is why the results do not include that orientation. Measurements were performed for temperatures of 25, 60, 80, 110, 140 °C in a frequency range 10^−1^ Hz to 2 × 10^7^ Hz.

## 3. Results and Discussion

### 3.1. Microscopic Analysis of Printed Structures

Samples of each printing direction were observed with an optical microscope with 20× magnification. As predicted, anisotropy of the mineral particles in material could be observed. Based on the observed images, the filler particles could be described as flake-shaped.

**Figure 2:**
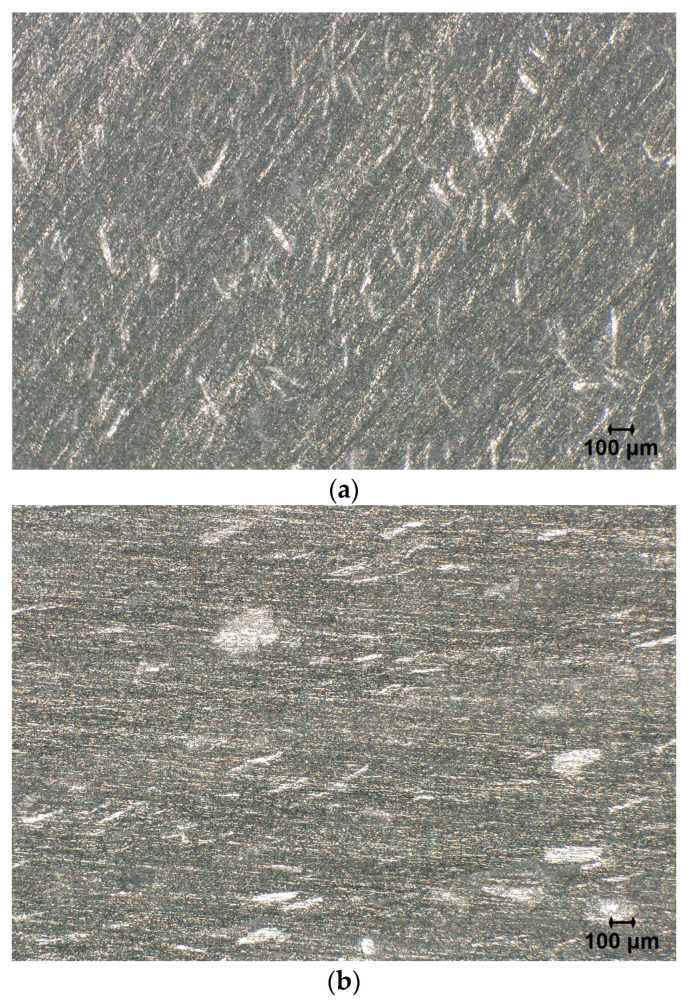

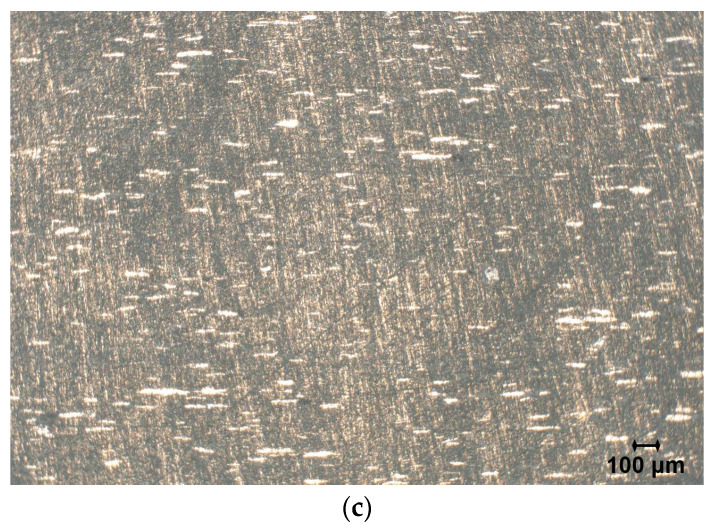
Microscopic images of the polished flat disc surfaces for: (**a**) sample X; (**b**) sample Y; and (**c**) sample Z. The microscopic observation area is marked in Figure 1.

In Figure 2a, the cross-section of the extruded lines is visible; the orientation of the particles is not dominated by a single direction. In Figure 2b, the direction of observation is identical to the direction of the nozzle during printing. In that direction, materials were squeezed by the nozzle, which led to the orientation of flakes in such a way that the flat surfaces of the flakes are parallel to the sample surface, which is visible in the image as bright areas. In Figure 2c, the observed image is parallel to the nozzle path during printing and to the direction of layer growth. In this image, the high orientation of the particles can be observed in the direction of the nozzle path. These observations confirm that the microstructure of samples made with FGF technology is highly anisotropic and could be controlled by appropriately designed printing paths and the direction of layer growth.

### 3.2. Results of Thermal Conductivity

The values of the measured thermal conductivity and thermal resistance are presented in Table 3. The given results are the steady-state values of the thermal conductivity though the thickness direction of the samples. The values are average and standard deviation of five readings.

Samples X1 and X2, with the fillers aligned parallel to the heat flow, exhibit mean thermal conductivity of 4.09 W/m·K. On the other hand, the samples Y1 and Y2, with perpendicular filler orientation to the heat flow, show mean thermal conductivity of 1.21 W/m·K. This represents a 238% increase in the thermal conductivity when transitioning from perpendicular to parallel orientation. Sample Z demonstrates intermediate performance at 2.11 W/m·K, falling between the two extremes. The thermal anisotropy ratio between thermal conductivity for samples X and Y was calculated as 3.4. This indicates that heat flows 3.4 times more effectively through the material when fillers are aligned with the heat flow direction compared to perpendicular alignment.

**Figure 3:**
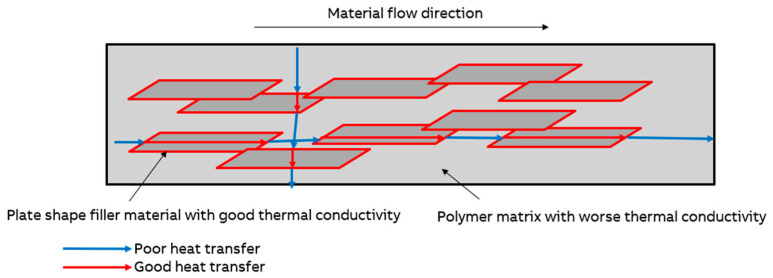
Schematic representation of the filler orientation and material flow during extrusion pellet printing and its effect on the heat transfer.

The highest thermal conductivity and the observed 238% enhancement in the thermal conductivity for sample X with the parallel filler orientation can be attributed to the formation of preferential thermal conduction pathways, as shown in Figure 3. When platelet-shaped or elongated mineral fillers align parallel to heat flow, they create continuous or near-continuous chains of the high-conductivity material, facilitating efficient phonon transport [30,31]. In contrast, the perpendicular orientation forces the heat to traverse multiple filler–polymer interfaces, each contributing interfacial thermal resistance (Kapitza resistance) that impedes the heat transfer [32].

The higher value for sample Z compared to sample Y can be explained by the fact that during the printing of sample Z, the printing nozzle pressed down on subsequent extruded layers, causing the majority of the plate-like filler to become oriented parallel to the direction of heat flow (see Figure 1 and Figure 2). However, during the printing of sample Y, the printing nozzle pressed down the extruded layers; additionally, the printing direction caused most of the plate-like mineral filler to be oriented transversely to the direction of heat flow.

The magnitude of anisotropy observed in this study (3.4) is consistent with that of studies in the literature for polymer composites containing platelet-shaped fillers, such as graphite, boron nitride, or mica, where anisotropy ratios ranging from 2 to 5 are commonly reported depending on filler aspect ratio, loading fraction, and degree of alignment [33,34]. Higher-aspect ratio fillers and more perfect alignment can yield even larger anisotropy ratios, exceeding 10 in some systems [35]. A comparison of the thermal conductivity and anisotropy ratio in different 3D-printed polymer composites is shown in Table 4.

In the case of thermal resistance, the values inversely correlate with the thermal conductivity (cf. Equation (2)). The parallelly oriented samples (X1, X2) exhibit mean thermal resistance of 24.8 × 10^−4^ m^2^·K/W, while perpendicularly oriented samples (Y1, Y2) showed values of 82.6 × 10^−4^ m^2^·K, which indicates a 70% reduction in thermal resistance achieved through proper filler alignment. This substantial difference has critical implications for thermal management design, as thermal resistance directly determines the temperature rise across the material under a given heat flux. The observed improvement can be translated directly to reduced operating temperatures in electronic devices and power systems. For example, for a typical heat flux of 10 W/cm^2^, the observed resistance difference corresponds to a temperature drop reduction of 58 °C, which is a highly significant improvement for component reliability and performance.

The substantial thermal anisotropy presented in this study has important implications for both material design and manufacturing process selection. For applications requiring maximum heat dissipation in a specific direction (e.g., through-plane heat transfer in thermal interface materials), manufacturing processes should be optimized to align fillers parallel to the primary heat flow path. The ability to engineer thermal anisotropy provides an additional degree of freedom in thermal management design beyond simply maximizing filler loading.

### 3.3. Dielectric Properties

**Figure 4:**
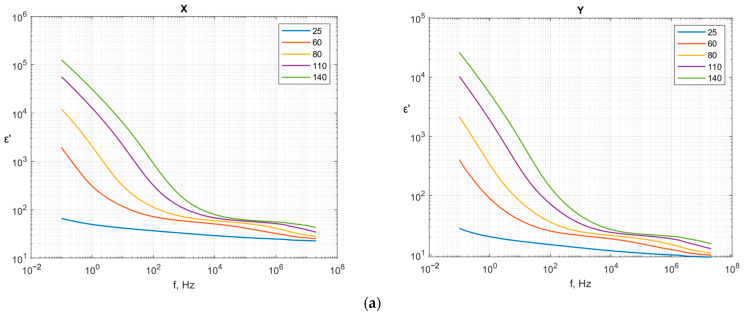

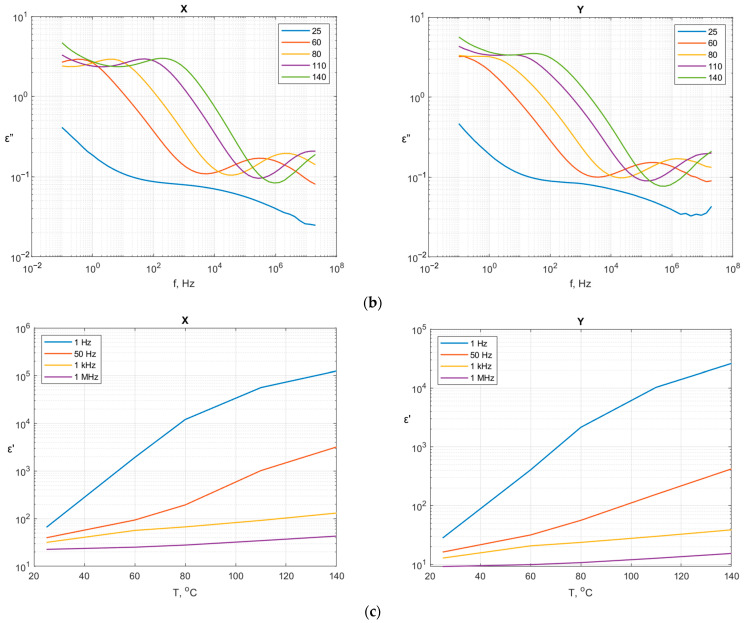
Dielectric results: (**a**) permittivity (ε′) as functions of frequency; (**b**) dielectric loss (ε″) as functions of frequency; and (**c**) permittivity (ε′) as functions of temperature for samples X and Y.

The values of permittivity (ε′) and dielectric loss (ε″) as functions of frequency and temperature for samples X and Y are presented in Figure 4. The dielectric measurements reveal significant anisotropy in the electrical properties of the 3D-printed structures, which correlates with the observed thermal anisotropy discussed earlier in Section 3.2. For both samples, significant increases in the dielectric constant with increasing temperatures are observed, particularly at lower frequencies. This behavior is characteristic of PA6 and its blends, where increased molecular mobility at elevated temperatures enhances dipolar relaxation processes [36,37]. The temperature dependence can be attributed to several mechanisms: (i) enhanced segmental mobility of the polymer chains, allowing for more effective dipole orientation under the applied electric field; (ii) increased ionic conductivity due to moisture absorption and impurity migration; and (iii) interfacial polarization at the filler–matrix boundaries, which becomes more pronounced at higher temperatures due to reduced interfacial energy barriers.

The permittivity shows typical dielectric dispersion, decreasing with increasing frequency across all temperatures. This frequency dependence follows the Debye-type relaxation behavior commonly observed in semicrystalline polymers. At low frequencies (<10^2^ Hz), interfacial polarization (Maxwell–Wagner–Sillars effect) dominates, resulting in higher apparent permittivity values. At higher frequencies (>10^5^ Hz), only electronic and atomic polarization mechanisms can respond to the rapidly alternating field, leading to reduced permittivity values.

Notably, the orientation presented in sample X is characterized by a higher dielectric constant and conductivity than sample Y across the entire frequency and temperature range. The dielectric constant for sample X is approximately 15–30% higher than sample Y at room temperature, with this difference becoming more pronounced at elevated temperatures. The dielectric permittivity anisotropy ratio (ca. 1.3) is lower than the thermal anisotropy ratio (3.4), suggesting that different structure–property relationships govern electrical and thermal transport

The observed dielectric anisotropy can arise from two potential mechanisms:

- Filler orientation effect: the platelet-shaped mineral fillers, when aligned parallel to the electric field direction (sample X), create more extensive interfacial regions perpendicular to the field, enhancing interfacial polarization. The flake-like morphology observed in the microscopic analysis (see Figure 2) supports this interpretation. Studies on mica and clay-filled polymer composites have reported similar orientation-dependent dielectric behavior, with anisotropy ratios ranging from 1.1 to 1.8, depending on the filler aspect ratio and loading [38,39];
- Matrix microstructure effect: the FGF printing process may induce preferential orientation of PA6 crystallites and chain alignment along the extrusion direction. Previous studies on FDM-printed PA6 composites have demonstrated that processing-induced molecular orientation can significantly affect dielectric properties, with aligned chains exhibiting higher polarizability along the orientation axis [36].

Based on the current results, it is not possible to definitively determine whether the observed dielectric anisotropy primarily results from mineral filler orientation or from processing-induced changes in the PA6 matrix microstructure. The relatively modest dielectric anisotropy (compared to thermal anisotropy) suggests that both mechanisms may contribute, with filler orientation playing a dominant role in thermal transport, while matrix microstructure influences dielectric response.

The dielectric loss (ε″) and AC conductivity exhibit similar anisotropic trends, with sample X showing higher values than sample Y. The conductivity increases with both temperature and frequency, following a power-law relationship characteristic of hopping conduction mechanisms in semicrystalline polymers. At elevated temperatures (>100 °C), the conductivity increase becomes more pronounced, likely due to enhanced ionic mobility and the onset of space–charge polarization near the glass transition region of the amorphous PA6 phase.

Despite the observed anisotropy, both orientations maintain sufficiently low dissipation factor (tan δ < 0.05 at 1 kHz and 25 °C), confirming their suitability for electrical insulation applications. The dielectric strength values (see Table 1) remain well above typical requirements for medium-voltage applications. However, the orientation-dependent dielectric properties should be considered in device design, particularly for applications involving high-frequency operation or elevated service temperatures, where dielectric losses can contribute to additional heating. Table 5 shows the comparison of dielectric properties obtained in the presented work with the BN-filled polymer composites obtained by different manufacturing methods.

The observed dielectric behavior is consistent with recent studies on 3D-printed PA6 composites. Yang et al. [36] reported similar temperature-dependent increases in permittivity for FDM-printed CNT/PA6 composites with fractal microstructures, attributing the behavior to enhanced interfacial polarization at elevated temperatures. Perdum et al. [37] investigated PA6-based hybrid composites and observed comparable frequency dispersion patterns, emphasizing the role of filler–matrix interfaces in determining dielectric response. However, these studies focused on carbon-based fillers with inherently higher conductivity, whereas the present work employs electrically insulating mineral fillers, enabling the combination of enhanced thermal conductivity with maintained electrical insulation, which is a critical requirement for the application of materials as electrical insulation with enhanced thermal conductivity.

## 4. Conclusions

This study demonstrates that pellet-based fused granulate fabrication (FGF) enables precise control of thermal anisotropy in polymer composites through manipulation of the filler orientation. The following key findings were established:

- Filler orientation dominates thermal transport—the parallel alignment of mineral fillers yields thermal conductivity of 4.09 W/m·K compared to 1.21 W/m·K for perpendicular orientation, representing a 238% enhancement. This demonstrates that manufacturing process control is critical for optimizing thermal performance;
- Strong thermal anisotropy was achieved—the anisotropy ratio of 3.4 is one of the highest reported for electrically insulating 3D-printed thermoplastic composites. This directional heat transfer capability enables targeted thermal management in confined spaces;
- A significant reduction in thermal resistance was obtained—parallel-oriented samples exhibited 70% lower thermal resistance (24.8 × 10^−4^ m^2^·K/W) compared to samples with a perpendicular orientation (82.6 × 10^−4^ m^2^·K/W). For a typical heat flux of 10 W/cm^2^, this translates to a 58 °C reduction in temperature rise, directly improving device reliability;
- Electrical insulation was maintained—despite the enhanced thermal conductivity, the material preserved excellent dielectric properties with low dielectric loss (tan δ < 0.05 at 1 kHz) and high surface resistivity (4 × 10^14^ Ω), meeting requirements for electrical insulation in medium-voltage applications;
- Industrial scalability is possible—the pellet-based FGF process enables direct use of industrial-grade polymer composites without intermediate filament production, reducing cost and expanding material options for thermal management applications in electrical devices.

These results establish pellet-based additive manufacturing as a viable route for producing electrically insulating thermal management components with engineered anisotropic heat transfer, addressing a critical need in power electronics, LED systems, and electrification devices.

## 5. Patents

Rzepecki Michal, Banaszczyk Jedrzej, Rybak Andrzej, Grecki Filip, Bahmani Amin. 2024. Method for manufacturing of a thermally conductive electrical component. EP4353449A1, April 17.

## Figures and Tables

**Table 1:** Typical properties of investigated PA 6 system.

Properties	Description or Value	Unit
Density	1.68	g/cm^3^
HDT, 0.45 MPa	203	°C
Flexural strength	105	MPa
Tensile strength	75	MPa
Tensile strain	1.1	%
Impact strength	9	kJ/m^2^
Thermal conductivity through-plane	1.2	W/m·K
Thermal conductivity in-plane	5.5	W/m·K
Surface resistivity	4·× 10^14^	Ω
Dielectric strength	7.2	kV/mm
Flame class rating, UL 94	V-0	

**Table 2:** Instrument settings and conditions.

Settings and Conditions	Value
Upper-plate temperature (°C)	30
Lower-plate temperature (°C)	10
Ambient temperature (°C)	20
Measurement type	Through thickness
Expected accuracy	Within 5%

**Table 3:** Density and pore filling percentage for investigated samples.

Sample Type	Sample Thickness ^1^ (mm)	Thermal Conductivity (W/m·K)	Thermal Resistance ($10^{-4}m^{2}\cdot K/W$)
X1	10.12	4.12 ± 0.11	24.6 ± 0.6
X2 ^2^	10.15	4.06 ± 0.04	25.0 ± 0.2
Y1 ^2^	9.85	1.20 ± 0.01	82.4 ± 0.9
Y2	10.16	1.23 ± 0.02	82.7 ± 1.3
Z	10.15	2.11 ± 0.03	48.1 ± 0.7

^1^ As measured with a caliper. ^2^ Sample surfaces were not fully parallel and were corrected with interface material, but the impact on the results from this issue can be neglected. Average sample thickness is reported.

**Table 4:** Comparison of thermal conductivity (TC) in different 3D-printed polymer composites.

Polymer Matrix	Filler Type	TC Max (W/m·K)	TC Min (W/m·K)	TC Anisotropy Ratio	Printing Method	Electrical Properties	Reference
PA6	Mineral (BN-type)	4.09	1.21	3.4	FGF	Insulating	This work
Flexible resin	BN platelets (20 wt.%)	0.73	0.51	1.4	VPP	Insulating	[24]
PLA	Carbon fiber	0.20	0.16	1.2	FFF	Conductive	[25]

**Table 5:** Comparison of dielectric properties in BN-filled polymer composites obtained by different manufacturing methods.

Polymer Matrix	Filler Type	Dielectric Constant @ 1 kHz	Dissipation Factor @ 1 kHz	Manufacturing Method	Reference
PA6	Mineral (BN-type)	6 ÷ 8	<0.05	FGF	This work
Flexible resin	BN platelets (20 wt.%)	8	0.2	VPP	[24]
Epoxy resin	BN platelets (30 wt.%)	4.5	<0.02	Vacuum casting	[14]

## Data Availability

The original contributions presented in this study are included in the article. Further inquiries can be directed to the corresponding author.
